# Effects of exercise, physical activity, and sports on physical fitness in adults with Down syndrome: A systematic review

**DOI:** 10.3934/publichealth.2024029

**Published:** 2024-04-23

**Authors:** Felipe Montalva-Valenzuela, Antonio Castillo-Paredes, Claudio Farias-Valenzuela, Oscar Andrades-Ramirez, Yeny Concha-Cisternas, Eduardo Guzmán-Muñoz

**Affiliations:** 1 Laboratorio de Fisiología del Ejercicio y Metabolismo (LABFEM), Escuela de Kinesiología, Facultad de Medicina, Universidad Finis Terrae, Santiago, Chile; 2 Grupo Investigación en Actividad Física y Salud Escolar (AFySE), Escuela de Pedagogía en Educación Física, Facultad de Educación, Universidad de Las Américas, Santiago, Chile; 3 Facultad de Ciencias para el Cuidado de la Salud, Universidad San Sebastián, Providencia, Chile; 4 Facultad de Educación y Ciencias Sociales, Entrenador deportivo, Universidad Andres Bello, Concepción, Chile; 5 School of Pedagogy in Physical Education, Faculty of Education, Universidad Autónoma de Chile, Talca, Chile; 6 Universidad Arturo Prat, Iquique, Chile; 7 School of Kinesiology, Faculty of Health, Universidad Santo Tomás, Talca, Chile

**Keywords:** Down syndrome, adults, exercise, physical activity, physical fitness

## Abstract

This systematic review aimed to analyze the effects of exercise, physical activity, and sports on physical fitness in adults with Down syndrome (DS). A literature search was conducted across four databases EBSCO, Scopus, Web of Science, and PubMed. The PRISMA guidelines were followed. The PEDro scale and the Cochrane risk of bias tool were used to assess the quality and risk of the studies, respectively. The protocol was registered in PROSPERO (code: CRD42023449627). Of the 423 records initially found, 13 were finally included in the systematic review, in which 349 adults with DS participated. 92% of the articles declared at least one significant difference post-intervention. The available evidence indicates that exercise, physical activity, and sports have a positive effect on some variables of physical fitness, especially strength, balance, body composition, cardiorespiratory fitness, flexibility, and functional capacity. Furthermore, it should be considered as an additional treatment or complementary therapy to improve the functionality and quality of life of adults with DS.

## 1. Introduction

Down syndrome (DS) is a condition caused by trisomy 21 and is the most common genetic alteration of intellectual disability [1],[2]. An estimated occurrence suggests that 1 out of every 790 births is affected by this condition in the United States [3]. Individuals with DS are commonly identified by delayed motor development and challenges in executing functional motor tasks, a well-documented observation [4]. Children with DS often exhibit characteristics such as hypotonia, ligamentous hyperlaxity, delayed muscle activation, and deficits in posture control [5]–[7]. In the same way, there is a high tendency to congenital heart problems, which is one of the most important causes of mortality and morbidity in this population, especially in countries where surgical interventions are not routinely offered [8]–[10]. Adults with Down syndrome tend to have negative alterations in health indicators and generally have higher rates of obesity than their typically developing peers [11]. They also have impaired physical fitness, expressed in lower strength [12] and aerobic capacity [13] compared to adults without Down syndrome. Furthermore, it has been reported that a large part of adults with DS are sedentary, and it is estimated that less than 10% of this population reaches the minimum recommendation for physical activity [14]–[17]. Likewise, this population has a predisposition to premature sarcopenia [18] and a higher percentage of adiposity [19], both high-risk factors and mortality in adults [20]. Therefore, negative changes at the level of health indicators have been widely reported, with low physical fitness and low participation in physical and sports activities being of concern.

Physical fitness refers to the capacity to engage the body's systems efficiently and effectively, fostering a state of well-being that enables us to carry out our daily activities with ease [21]. This concept encompasses different physical abilities, among which are: Strength, speed, resistance, or cardiorespiratory endurance, flexibility, body composition, and balance [22],[23]. In contemporary times, physical fitness holds significant importance as a marker of overall health [24],[25], serving as a predictor for all-cause mortality [26],[27]. Additionally, enhancing cardiovascular and muscular fitness emerges as a viable strategy for improving overall health [28]. Specifically, at the muscular level, an increased capacity for push-ups is associated with a reduced incidence of cardiovascular disease [29]. Furthermore, lower handgrip strength has been linked to conditions such as dyslipidemia, hypertension, and type II diabetes [30],[31].

It is known that exercise generates multiple health benefits [32], including benefits in cancer prevention, cardiovascular health, musculoskeletal health, metabolic health, and neurocognitive health [33]. On the other hand, in children and adolescents with intellectual disabilities, improvements have been seen in health from resistance training [34],[35], in cardiorespiratory fitness from aerobic exercise [36], and in body composition [37],[38]. In addition, there are already reviews on the benefits of exercise in the population with DS, specifically in children and adolescents, where improvements in balance are mentioned [39], in motor performance [40], strength, and posture [41]. Also, improvements in health have been seen from exercise in adults with DS, specifically, improvements in cardiometabolic risk, muscle strength, and aerobic work capacity [42],[43], however, there are doubts regarding the intensity and frequency of its prescription. On the other hand, the literature on fitness in adults with DS is limited.

The analysis of this information can be useful to organize and confirm the benefits of exercise, physical activity, and sport on physical fitness in adults with DS. Considering the background presented, the present systematic review aims to analyze the effects of exercise, physical activity, and sports on physical fitness in adults with DS.

## 2. Materials and methods

### 2.1. Protocol and registration

The Preferred Reporting Items for Systematic Reviews and Meta-Analyses (PRISMA) guidelines [44] were used in this systematic review. PROSPERO provided registration and approval for the protocol (code: CRD42023449627).

### 2.2. Eligibility criteria

For this systematic review, the inclusion criteria were the following: (i) Randomized controlled trials (RCTs) or quasi-experimental clinical trials that used exercise as an intervention; (ii) the aim to improve some component of the physical fitness in people with DS; and (iii) people over than 18 years of both genders. In addition, studies were incorporated into the systematic review using the PICO framework (population: Adults with DS; intervention: exercise, sports, or physical activity; comparator: control group; outcomes: Some aspect of physical fitness).

The exclusion criteria were: (i) Cross-sectional, retrospective, and prospective studies, or whose interventions were not focused on physical activity, exercise, or sport; (ii) studies with a co-intervention, such as medications, nutritional supplements, or an educational program; (iii) non-original articles (for example, translations, book reviews, letters to the editor); (iv) duplicate articles; (v) review articles (e.g., narrative reviews, systematic reviews, meta-analyses); and (vi) case studies.

### 2.3. Data sources and searches

The search process was carried out in July 2023, using four databases: EBSCO, Scopus, Web of Science, and PubMed. The articles used in this review were obtained without restrictions on language or publication year up until the extraction date in July 2023. The search string used was the following: ((“Adult” OR “Adults”) AND (“Down syndrome”) AND (“Physical activity” OR “Exercise” OR “physical exercise” OR “Sports”) AND (“Physical fitness” OR “Fitness”)).

### 2.4. Study selection

Rayyan web software (http://rayyan.qcri.org) was used by two independent reviewers (FM-V and EG-M) for the study selection process [45]. After removing duplicates, studies were selected by title and abstract. Subsequently, the same reviewers applied the inclusion and exclusion criteria to evaluate the full text of potentially eligible articles. In case of disagreement during the study selection phase, a third author was consulted to reach a final decision (YC-C).

### 2.5. Data collection

Data were extracted from the studies by 2 independent reviewers (FM-V and EG-M). In case of discrepancy, a third review author (YC-C) compared the extracted data and resolved the disagreement. Data collected for each study included author, year of publication, country, sample size, age, data collection, intervention characteristics (frequency and duration of sessions), control intervention, and major outcomes. The authors were contacted by e-mail to obtain information if relevant data were not included in the study.

### 2.6. Methodological quality assessment and risk of bias

The chosen studies underwent assessment using the PEDro scale [46],[47]. This tool measures the methodological integrity of the research, scrutinizing 11 aspects such as the blinding process, statistical evaluation, details on randomization, and the representation of results in the research being assessed. The first criterion estimates the external validity and is not factored into the final outcome. Criteria 2 through 11 consider the internal validity of the paper, applying a standardized scoring system (ranging from 0 to 10). The quality of the study was categorized as excellent (9–10 points), good (6–8 points), fair (4–5 points), and poor (less than 4 points). This procedure was independently executed by two authors (FM-V and EG-M), and a third reviewer (AC-P) resolved any discrepancies between the reviewers.

The Cochrane Risk of Bias (RoB-2) tool for randomized clinical trials was used to assess the risk of bias in the included trials. The assessment was carried out independently by two reviewers (FM-V and EG-M). Each domain (randomization process, departures from the intended interventions, missing outcome data, outcome measurement, selection of the reported outcome, and overall) was rated as “low”, “some concerns”, or “high” risk of bias [48].

### 2.7. Data synthesis

The subsequent data was collected and scrutinized from the selected studies: (i) The names of the authors and the year of publication; (ii) the geographical location where the research was conducted; (iii) the design of the study; (iv) the initial health status of the sample; (v) the count of participants in both the intervention and control groups, along with the percentage of women; (vi) the average age of the sample group; (vii) the tasks undertaken in both the experimental (EG) and control groups (CG), as well as the volume of training (total duration, frequency per week, and time per session); (viii) the tools used for collecting data on physical performance; and (ix) the major findings of the studies.

## 3. Results

### 3.1. Studies selection

**Figure 1.**
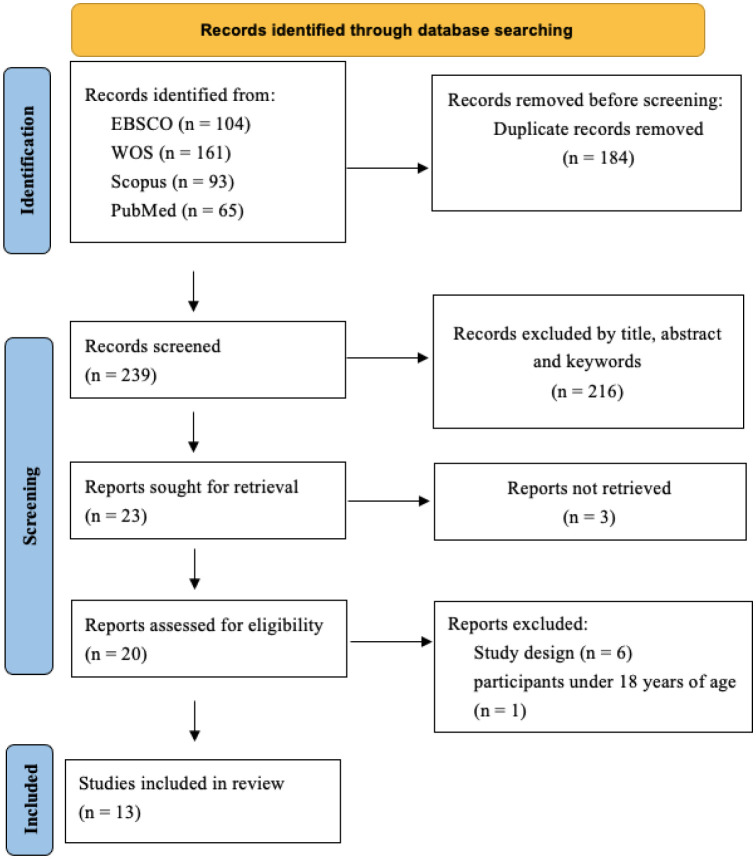
PRISMA flowchart of the search strategy and study selection.

In the identification phase of the study, 423 articles were found. Then, duplicates were removed through Rayyan (n = 184) and studies were filtered through title, abstract, and keywords (n = 216), obtaining 23 articles for retrieval. In the analysis phase, 3 studies were excluded because the full text was not available. In the analysis of 20 studies, 6 were excluded based on their study design, and an additional one was excluded because the participants were under 18 years old. Following this process, a total of 13 studies met all the requirements to be included in the review (Figure 1).

The PEDro scale was used to evaluate the 13 selected studies (Table 1). All the studies achieved a score equal to or greater than four points on the scale and were classified as fair: 4/10 [50], 5/10 [53],[56],[58],[60],[61], good: 6/10 [49],[52],[55],[59], 7/10 [54], and 8/10 [51],[57]. No studies of excellent methodological quality were found.

**Table 1.** Study quality assessment according to PEDro scale.

**Authors**	**Criteria**											
	**1**	**2**	**3**	**4**	**5**	**6**	**7**	**8**	**9**	**10**	**11**	**Total**
Boer, 2020 [49]	Y	Y	N	Y	N	N	N	Y	Y	Y	Y	6
Boer & deBeer, 2019 [50]	Y	N	N	Y	N	N	N	Y	N	Y	Y	4
Boer & Moss, 2016 [51]	Y	Y	N	Y	Y	N	Y	Y	Y	Y	Y	8
Cai & Baek, 2022 [52]	Y	Y	N	Y	N	N	N	Y	Y	Y	Y	6
Cowley et al., 2011 [53]	Y	N	N	Y	N	N	N	Y	Y	Y	Y	5
Diaz et al., 2021 [54]	Y	Y	Y	Y	N	N	N	Y	Y	Y	Y	7
Perrot et al., 2021 [55]	Y	Y	N	Y	N	N	N	Y	Y	Y	Y	6
Rimmer et al., 2004 [56]	Y	Y	N	Y	N	N	N	Y	N	Y	Y	5
Shields et al., 2008 [57]	Y	Y	Y	Y	N	Y	N	Y	Y	Y	Y	8
Shin et al., 2021 [58]	Y	N	N	Y	N	N	N	Y	Y	Y	Y	5
Silva et al., 2017 [59]	Y	Y	N	Y	N	Y	N	Y	N	Y	Y	6
Tsimaras et al., 2003 [60]	Y	N	N	Y	N	N	N	Y	Y	Y	Y	5
Varela et al., 2001 [61]	Y	N	N	Y	N	N	N	Y	Y	Y	Y	5

Note: Y = Yes; N = No. The following are the criteria for the PEDro scale: 1: The selection criteria were specified (not included in the total score). 2: The distribution of participants to the groups was random. 3: The task was concealed. 4: The groups were similar in terms of the most relevant predictive factors. 5: All participants were blinded. 6: The therapists involved in the intervention were all blinded. 7: Assessors who measured at least one significant outcome were blinded. 8: At least 85% of the key results were achieved. 9: All outcomes for participants who underwent the intervention were reported, for at least one main result. 10: A statistically significant difference was found between the groups for at least one primary outcome. 11: The intervention showed point measures and variability for at least one main outcome. The study quality was categorized as excellent for 9–10 points, good for 6–8 points, fair for 4–5 points, and poor for less than 4 points.

### 3.2. Risk of bias

**Figure 2.**
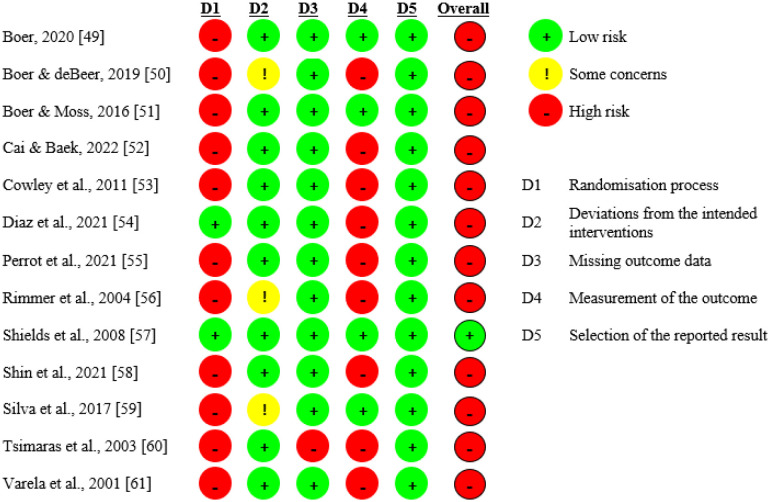
Assessment of risk of bias based on the Cochrane risk of bias tool.

**Figure 3.**
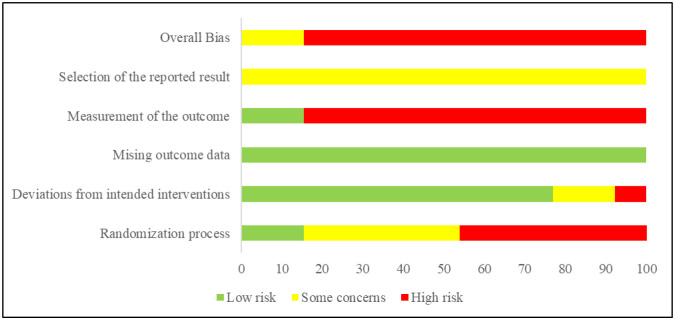
Risk of bias graph.

Figure 2 displays the information concerning the bias risk associated with the studies incorporated. The results of the analysis showed that 12 of the 13 studies included presented a high risk of selection bias and one was categorized as low risk. Figure 3 shows the percentage obtained by judgment in each evaluated item (low risk, some concerns, high risk).

### 3.3. Studies characteristics

Table 2 gives a summary of the variables that were examined in each of the studies chosen. Of these, three were performed in South Africa [49]–[51], one in China [52], two in USA [53],[56], one in Spain [54], one in France [55], one in Australia [57], one in Korea [58], two in Portugal [59],[61], and one in Greece [60]. Concerning the design of the studies, nine were randomized controlled trials and four were not randomized controlled trials.

**Table 2.** Studies reporting on the effect of exercise, physical activity, and sports on the physical fitness of adults with DS.

Study	Country	Study design	Groups (n) and Sample size Female (%)	Mean age (year)	Intervention		Data collection instruments of physical fitness	Main outcomes
					Experiment group (EG)	Control group (CG)		
Boer, 2020 [49]	South Africa	RCT	26 EG: 13 CG: 13 50 % Female	34.2 30.3	Aquatic training 3 × 30 min/week 8 weeks	Usual activities	Body mass, BMI, 16-m PACER (shuttles), 6 MWD (m), Standing on one leg (s), Walking on balance beam (steps), 8-ft up and go (s), Sit-to-stand (s), Modified curl-up (n), Isometric push-up (s), 12-m swim time (s), 24-m swim time (s)	EG: ↓ Body mass; ↓ BMI; ↑ 16-m PACER (shuttles); ↑ 8-ft up and go, ↑ Modified curl-up (n); ↑ Sit-to-stand (s); ↑ Isometric push-up (s); ↑ 12-m swim time (s). EG vs CG: significant differences in favor EG.
Boer & deBeer, 2019 [50]	South Africa	NRCT	23 EG: 13 CG: 10 43.7% Female	31.4 31.1	Aquatic training 3 × 35 min/week 6 weeks	Usual activities	BMI, 16-m PACER (shuttles), 6 MWD (m), Standing on one leg (s), Walking on balance beam (steps), 8-ft up and go (s), Sit-to-stand (s), Modified curl-up (n), Isometric push-up (s)	EG: ↑ 16-m PACER (shuttles), ↑ 6 MWD (m), ↑ Sit-to-stand (s), ↑ Modified curl-up (n). EG vs CG: significant differences in favor EG.
Boer & Moss, 2016 [51]	South Africa	RCT	42 EG (IT): 13 EG (CAT):13 CG:16 40.4% Female	30.0 34.2 36.6	IT 3 × (10–30 s all out sprints with 90 s of rate) × week 12 weeks CAT 3 × 30 min/week 12 weeks	Usual activities	Body mass(kg), Waist circumference (cm), Fat mass (kg), Peak VO2 (L/min), Rel. peak VO2 (mL/kg/min), VE (L/min), Time to exhaustion (s), 6 MWD (m), 8-ft up and go (s), Sit-to-stand (amount/30 s)	CAT: ↑ 8-ft up and go (s), ↑ Sit-to-stand (amount/30 s), ↑ 6 MWD (m), ↑ Time to exhaustion (s), ↑ Peak VO2 (L/min), Weight (kg) ↓. IT: ↑ Peak VO2 (L/min), ↑ VE (L/min), ↑ Time to exhaustion (s), Weight (kg) ↓. IT/CAT vs CG: significant differences in favor IT/CAT.
Cai & Baek, 2022 [52]	China	RCT	22 EG: 11 CG: 11 18,1% Female	All 24.7	Basketball program 3 × 60 min/week 24 weeks	Usual activities	Body mass, BMI, Waist circumference (cm), 16-m PACER (shuttles), Modified curl-up (n), Standing on one leg (s), one-minute single-handed dribble (n), One-minute shot (n), Sit-and-reach test (cm)	EG: Body mass ↓, BMI ↓, Waist circumference (cm) ↓, 16-m PACER (shuttles) ↑, Modified curl-up (n) ↑, Standing on one leg (s) ↑, one-minute single-handed dribble (n) ↑, One-minute shot (n) ↑, Sit-and-reach test (cm) ↑. EG vs CG: significant differences in favor EG.
Cowley et al., 2011 [53]	USA	NRCT	30 EG: 19 CG: 11 43.3% Female	29 27	Progressive resistance training 2 × week 10 weeks	Usual activities	ISOK and ISOM KE, KF, PT, Time to ascend 10 steps (s), Time to descend 10 steps (s), Absolute peak VO2 (ml/min), Relative peak VO2 (ml/min/kg)	Time to ascend 10 steps (s) ↑, Time to descend 10 steps (s) ↑, Relative peak VO2 (ml/min/kg) ↑, ISOM KE PT at 75° (N/m) ↑, ISOM KE PT at 60° (N/m) ↑, ISOM KE PT at 45° (N m) ↑. EG vs CG: significant differences in favor EG.
Diaz et al., 2021 [54]	Spain	RCT	36 EG: 18 CG: 18 Not reported	All 28.1	Resistance training program 3 × week 12 weeks	Usual activities	Body mass(kg), BMI (kg/m2), MM (kg), SMI (kg/m2), CK, Mb, LDH	EG: MM (kg) ↑, SMI (kg/m2) ↑. EG vs CG: significant differences in favor EG.
Perrot et al., 2021 [55]	France	RCT	12 EG: 6 CG: 6 50% Female	49.3 51.4	Exergame training 2 × 60 min/week 12 weeks	Usual activities	Cognitive Changes, Corsi block tapping, Stimulus Barrage Test, Stimulus Barrage Test, Physical Changes, Timed Up and Go, Timed Up and Down Stairs, 30-second Chair Stand, 6 MWD (m)	Timed Up and Go ↑, Timed Up and Down Stairs ↑, 30-second Chair Stand ↑, 6 MWT (m) ↑. EG vs CG: significant differences in favor EG.
Rimmer et al., 2004 [56]	USA	RCT	52 EG: 30 CG: 22 55.7 % Female	38.6 40.6	Exercise program 3 × 45 min/week 12 weeks	Usual activities	Peak VO2, Peak heart rate, Time to exhaustion, Bench press, Leg press, Hand grip left, Hand grip right, Body mass, BMI	Peak VO2 ↑, Peak heart rate ↑, Time to exhaustion ↑, Bench press ↑, Leg press ↑, Body mass↓. EG vs CG: significant differences in favor EG
Shields et al., 2008 [57]	Australia	RCT	20 EG: 9 CG: 11 35 % Female	25.8 27.6	Progressive resistance training program 2 × week 10 weeks	Usual activities	Chest press 1-RM (kg), Leg press 1-RM (kg), Chest press endurance (no. of repetitions), Leg press endurance (no. of repetitions), Timed up and down stairs test (s), Grocery shelving task (s)	Chest press endurance (no. of repetitions) ↑. EG vs CG: significant differences in favor EG.
Shin et al., 2021 [58]	Korea	NRCT	20 EG: 10 CG: 10 25 % Female	All 44.55	Resistance and balance training program 3 × 60 min/week 8 weeks	Usual activities	Body mass, BMI, FFM, % FAT Waist, Sit-to-Stand (n), Muscle Strength (kg), ISWT (sec), 5 m walking (sec)	Body mass ↓, Body mass index ↓, Body fat percentage ↓, Waist circumference ↓. EG vs CG: significant differences in favor EG.
Silva et al., 2017 [59]	Portugal	RCT	25 EG: 12 CG: 13 Not reported	aged between 18 and 60 years	Wii-based exercise program 3 × 60 min/week 8 weeks	Usual activities	Body composition: body mass, BMI, body fat %, visceral fat, muscle mass, waist circumference Fitness: test speed of limb movement, handgrip test, sit and reach, test flexibility. Motor efficiency: ruininks–Oseretsky response Speed	Body fat % ↓, Muscle mass ↑, Waist circumference ↓, Handgrip Test ↑, Ruininks–Oseretsky response Speed ↑, Sit-and-reach test (cm) ↑. EG vs CG: significant differences in favor EG.
Tsimaras et al., 2003 [60]	Greece	NRCT	25 EG: 15 CG: 10 Not reported	24.5 24.7	Aerobic training program 3 × 60 min/week 12 weeks	Usual activities	Heart Rate peak VE peak VO2 peak	VE peak ↑, VO2 peak ↑. EG vs CG: significant differences in favor EG.
Varela et al., 2001 [61]	Portugal	RCT	16 EG: 8 CG: 8	22 20.8	Rowing exercise regimen 3 × week 16 weeks	Usual activities	Heart Rate peak VE peak VO2 peak Respiratory exchange ratio	No significant differences

Note: EG: experimental group; CG: control group; Usual activities: routine life without intervention; IT: interval training; CAT: continuous aerobic training; BMI: body mass index;; m: meter; cm: centimeters; ml: milliliter; m: minute; n: repetitions; s: seconds; kg/kilogram; NRS: not reported separately; NRCT: non-randomized controlled trial; RCT: randomized controlled trial; 6 MWD: 6-minute walk distance; HGS; hand grip strength; Ve peak: peak minute ventilation; VO2 peak: peak oxygen consumption; Rel peak VO2: relative peak VO2; L: Liter; ISOK: isokinetic; ISOM: isometric; KE: knee extensor; KF: knee flexor; PT: peak torque; N/m: newton/meter; MM: muscle mass; SMI: skeletal muscle index; CK: creatine kinase activity; Mb: myoglobin concentration; LDH: lactate dehydrogenase activity; FAT: body far percent; FFM: fat free mass; ISWT: 10 m incremental shuttle walking test; ↑: significant increase (p ≤ 0.05); ↓: significant decrease (p ≤ 0.05).

### 3.4. Physical fitness outcomes and collection instruments

#### 3.4.1. Body composition

Body composition was evaluated by 8 studies through: weight, body mass index, fat mass, % body fat, waist circumference, and muscle mass [49]–[52],[54],[56],[58],[59]. 4 studies use a “Seca scale” and “stadiometer” [49]–[52], 4 studies use a bioelectrical impedance for body composition [51],[54],[58],[59], and one uses a skinfold caliper [56].

#### 3.4.2. Cardiorespiratory fitness

Cardiorespiratory fitness was assessed by 8 studies. It was evaluated through the heart rate peak, VE peak, VO2 peak, time to exhaustion [51],[53],[56],[60],[61], and aerobic capacity was measured through 16-m PACER, 6-minute walk distance [49],[50],[55].

#### 3.4.3. Strength

Muscle strength was assessed by 7 studies [49],[50],[53],[56]–[59]. 3 studies examined muscle strength using a dynamometer [53],[56],[58], 1 through exercise repetition maximum (RM) [57], 1 through the Eurofit test battery [59], 1 through the modified curl-up [49], and the last one using the isometric push up test [50].

#### 3.4.4. Functional capacity, flexibility, and balance

Functional capacity, flexibility, and balance were assessed by 8 studies [49]–[52],[55],[57]–[59]. It was measured through different tests, among them, standing on one leg (balance), walking on a balance beam (balance), 8-ft up and go (functional capacity), sit-to-stand (functional capacity), sit-and-reach test (flexibility), timed up and go (functional capacity), timed up and down stairs (functional capacity), grocery shelving task (functional capacity), and ruininks-oseretsky response speed (functional capacity).

### 3.5. Interventions

All the studies had two analysis groups, the EG, whose participants carried out the exercise intervention, and the CG, whose participants carried out their usual activities. Table 3 summarizes the exercise, physical activity, or sports protocol used in the included studies.

### 3.6. Main outcomes

The results of this systematic review showed that exercise, physical activity, and sports have beneficial effects on physical fitness in adults with DS. Of the 13 articles included in the review, 12 of them reported significant changes in favor of the EG in at least one variable related to physical fitness compared to CG.

Improvements in body composition, muscle strength, balance, flexibility, aerobic capacity and functional capacity [49],[51],[52],[55],[58],[59], upper body strength and balance [50],[57], leg strength [53], muscle mass and work task [54], all body strength [56], and cardiorespiratory fitness [60].

**Table 3.** Exercise, physical activity, or sport protocol used in included studies.

	Intervention protocol
Boer, 2020 [49]	Warm-up (4 min): walking in a circular motion inside the pool (1 min), marching in place whilst swinging the arms (1 min), few simple stretches (single-arm crossover, chest stretch, hamstring, calf, and quad stretch) (2 min). Preparation for the main session (6–7 minutes): two intervals of high intensity running on the spot 2 × (1-min interval, 30-s rest), one set of lunge jumps 45 s (15-s rest), one set of squat jumps 45 s (15-s rest), flutter kicks whilst holding onto the side of the pool 1 min (30-s rest). Main session (20 min): repetitive freestyle swim training (17 min), swimming lengths whilst holding onto the kicking board (3 min).
Boer & deBeer, 2019 [50]	Warm-up (5 min). Main session: arm circles, side twists, walk in place, run in place, water scoops, side leg lift, flutter kick on back, flutter kick on stomach, jumping jacks, knee twists, side shuffle, squat jumps, lunge jumps and a longer jog in place (35–45 min). Cool down (2 min).
Boer & Moss, 2016 [51]	Interval training: Warm-up (5 min). Main session: 10–30 s all-out sprints with 90 s (1:3 work-rest ratio) (20 min). Cool down (5 min). continuous aerobic training: Warm-up (5 min). Main session: cycling or walking at an intensity of 70% to 80% of VO2 peak (20 min). Cool down (5 min).
Cai & Baek, 2022 [52]	Warm-up: with various games (10 min). Main session: basic basketball skill learning (shooting, passing, and handling) and physical training (45 min). Cool down (5 min).
Cowley et al., 2011 [53]	Warm-up: not reported. Main session: three sets of 8–10 repetitions of leg extension and flexion, leg press, shoulder press, chest press, bicep curl and triceps pushdown performed using exercise machines. Cool down: not reported.
Diaz et al., 2021 [54]	Warm-up (10 min). Main session: six stations: arm curl (elbow flexion), triceps extension (elbow extension), leg extension, seated row, leg curl (knee flexion), and leg press (combined hip and knee extension), all exercise, based on the 8-repetition-maximum test. Cool down (10 min).
Perrot et al., 2021 [55]	Warm-up (time not reported). Main session: Wii sports (Wii Tennis and Wii Bowling) and Wii Fit Plus using the balance board to play Wii Soccer Headers, Wii Ski Jump, Wii Hula Hoop, and the Wii Marbles games). Cool down (time not reported).
Rimmer et al., 2004 [56]	Warm-up (3–5 min). Main session: cardiovascular exercise: treadmill, and elliptical cross-trainer (30–45 min) and muscular strength and endurance: bench press, seated leg press, seated leg curl, triceps push-down, seated shoulder press, seated row, lat pull-down, and biceps curl (15–20 min) Warm-up (3–5 min).
Shields et al., 2008 [57]	Warm-up (not reported). Main session: 6 exercises using weight machines: 3 for the upper limbs (shoulder press, seated chest press, seated row) and 3 for the lower limbs (seated leg press, knee extension, seated calf raises). Cool down (not reported).
Shin et al., 2021 [58]	Warm-up (10 min). Main session: upper muscle strength program with an elastic band (15 rep each), internal & external rotation exercise, chest press, standing row, crawling (10 m shuttle). Lower muscle strength program (15 rep each), sit-to-stand, calf raises against the wall, side lunge, rabbit jump and trampoline jump. Balance program, walking on the heel, kick to front & back (15 repetitions each), bounce on a gym ball, touch the left and right bottom with hands while sitting on a gym ball. Cool down (10 min).
Silva et al., 2017 [59]	Warm-up (not reported). Main session: Wii Fit Balance Board (“free run”, “heading”, “table tilt”, “snowboard slalom”, “tightrope tension”, “hula hoop”, “balance bubble” and “penguin slide”) and Wii Sports, Wii Sports Resort, Wii Fit and Just Dance 2). Cool down (not reported).
Tsimaras et al., 2003 [60]	Warm-up: light jogging followed by a round of light calisthenics (10–15 min). Main session: jogging and walking within the exercise area at different intensities (30 min). Warm-up (5 min).
Varela et al., 2001 [61]	Warm-up (10 min). Main session: rowing ergometer 55 to 70% of their peak VO2. Cool down (10 min).

## 4. Discussion

Our objective of this systematic review was to analyze the effects of exercise, physical activity, and sports on fitness in adults with DS. The major findings show that exercise, physical activity, and sports have a positive and significant effect on some variables of physical condition, especially on strength, balance, body composition, cardiorespiratory fitness, flexibility, and functional capacity.

Of the 13 articles included in the review, eight based their intervention on exercise, two on physical activity, and three on sports programs. Specifically, one based on rowing [61], one based on aerobic training [60], five on resistance training [53]–[58], one was divided into an interval group, and the other group in continuous aerobic [51], two they used exergaming as an intervention method [55],[59], two aquatic sports programs [49],[50], and one a basketball program [52].

Concerning intervention times, three studies carried out 8 weeks [49],[58],[59], one study carried out 6 weeks [50], two studies carried out 10 weeks [53],[57], five studies used 12 weeks of intervention [51],[54]–[56],[60], one study used 16 weeks [61], and one used 24 weeks of intervention [52]. Although there is heterogeneity around the types of intervention, there is a certain consensus on the weekly frequency and the minimum time of exercise, 2 or 3 weekly sessions with a minimum of 30 minutes, for at least 6 weeks to see significant effects on physical fitness.

### 4.1. Body composition

The results show improvements in some indicators of body composition, such as weight, body mass index, waist circumference, percentage of body fat, and muscle mass [49],[51],[52],[54],[56],[58],[59]. Although significant results were observed in this review, there is difficulty in achieving optimal results from exercise in this population [62]. Rossato et al. [63] mention that not all fat percentage equations are valid for people with DS and González-Agüero et al. [64] propose another body composition equation in this population, which could justify the ambiguity of some results. Seron et al. [65] mention that 2 different training programs (aerobic training and resistance training) did not generate improvements in fat percentage in adolescents with DS, and Diaz et al. [54] observed that there are no improvements in the percentage of fat, but there are improvements in the percentage of muscle mass from resistance training. Therefore, it is important to continue working and studying this variable in this population since body composition is a good indicator of health [66] and quality of life [67]. Being even more specific, the percentage of fat should be the main objective to continue working on since no consistent improvements have been observed, unlike the percentage of muscle mass, which has obtained significant improvements [68].

### 4.2. Cardiorespiratory fitness

Concerning cardiorespiratory fitness, significant improvements were observed in two parameters, maximum VO2 and maximum VE [49],[51],[56],[60]. Both parameters are considered a protective factor for various events of cardiovascular mortality and healthy aging [69],[70]. These improvements in cardiorespiratory fitness parameters could be due to a greater expression of messenger RNA, responsible for encoding proteins generally located in mitochondria, which are associated with higher levels of VO2 and mitochondrial respiration [71]; however, to conclude this, a more in-depth study would be necessary. Despite what was mentioned above, these results are important, since there is controversy with the adaptations that can be achieved in this population, Bahiraei et al. [72] mention that some exercise interventions may not achieve cardiorespiratory adaptations, mainly when looking for improvements in heart rate and VO2 maximum, arguing that the duration and intensity of the session are the main reasons for the difference in results. Furthermore, Beck et al. [73] conclude that individuals with DS present cardiovascular, ventilatory, and muscular deterioration of the cardiopulmonary system, which could justify the difficulty of achieving adaptations in this population.

### 4.3. Strength

The results mention significant effects on strength, specifically in the hand grip, knee extension torque, and the number of repetitions in bench press, shoulder press, leg press, modified curl-up, and isometric push-ups [49],[53],[56],[57],[59], important indicators since strength is a marker of physical condition, general health, and prevention of mortality from all causes [74], in addition, the development of strength is a protective factor against sarcopenia [75], a risk factor in this population. These results corroborate what was mentioned by Melo et al. [12] and Sugimoto et al. [76] and confirm that strength training is safe in this population. These significant changes could be justified due to neural adaptations (intramuscular and intermuscular coordination) and muscular adaptations (hypertrophy) [77] or by an increase in the activation rate of motor units [78].

### 4.4. Functional capacity, flexibility, and balance

Improvements were observed in different tests on functional capacity: 8-ft up and go, sit-to-stand, timed up and go, timed up and down stairs, Ruininks-Oseretsky response speed [49]–[54]. These findings are important since functional capacity is an important element within routine activities [79] and adults with DS have a decreased functional capacity, mostly because of high levels of obesity and low levels of cardiovascular fitness and muscle strength [80]. However, it is necessary to continue investigating this area in this population, since the main results observed are in children with DS, where it has been seen that their quality of life is affected by functional capacity [81].

In relation to flexibility, the review by Rodríguez-Grande et al. [82], mentions that there is no evidence on improvements in flexibility and that it is not a main result to measure; however, the present review does declare improvements in flexibility, specifically, in the sit and reach test. This improvement could be justified by the adaptations generated from resistance exercise in the range of motion [83]. On the other hand, one of the reasons why improved flexibility is not considered as a main outcome could be the hypotonia of people with DS, which is associated with lower muscle strength, greater laxity and therefore, better flexibility, so it would not be necessary to seek further adaptations.

Also, the conclusions of Guerrero et al. are corroborated. [84] where significant improvements in balance are mentioned in this population, these results could be due to the instability generated by exercise, which must be compensated by the body [85].

These results corroborate the importance of promoting exercise, physical activity, and sport in this population, given that the level of physical fitness is a fundamental factor that promotes well-being and a better quality of life [86],[87]. Although there are reviews that declare improvements in aspects of strength and functional capacity from some types of physical activity, such as exergaming [88], and suggestions of at least two days of resistance training per week for health benefits in this population [12], there is confusion about cardiorespiratory fitness and body fat percentage [62].

Adults with DS have lower cardiorespiratory responses than neurotypical adults [13], so exercise interventions related to this objective are necessary. However, it is important to always take congenital heart disease into account in this population when prescribing exercise. Finally, people with DS have different degrees of intellectual disability, from mild to severe, which can cause problems when learning the exercises [12], an aspect to consider when time to recommend and prescribe exercises in this population.

### 4.5. Limitations and strengths

Among the strengths of this review, it is found that all studies reported having a control group. Likewise, the tests used to collect data on fitness were similar, which allows the results to be better analyzed. Although there is heterogeneity around the intervention protocols, there is some similarity around the session time and weekly frequency. Among the limitations of this review is the heterogeneity of exercise interventions, where since the protocols are so different from each other, it is difficult to determine which intervention is more effective in time and results. The IQ of the participants and their nutritional status were also not considered, and studies on childhood and adolescence were excluded. Likewise, the role of the family within the interventions was not considered. Finally, the results of the present review were not presented according to sex. In future studies, it is advisable to factor in the physical activity levels of adults with Down syndrome and to review their habits and nutritional aspects while assessing body composition, particularly when examining body fat percentage. On the other hand, it would be extremely interesting to try another type of exercise intervention, for example, HIIT, multicomponent exercise program, or plyometric training, always considering the cardiac difficulties of this population.

## 5. Conclusions

The evidence collected indicates that exercise, physical activity, and sport have a positive and significant effect on fitness in adults with DS, specifically on strength, balance, body composition, cardiorespiratory fitness, flexibility, and functional capacity. Therefore, it is a concrete proposal that should be considered as an additional treatment or complementary therapy to improve the functionality and quality of life of adults with DS. Specifically, 2 or 3 sessions per week with a minimum duration of 30 minutes, for at least 6 weeks, generate benefits in the physical condition of adults with DS.

## Use of AI tools declaration

The authors declare they have not used Artificial Intelligence (AI) tools in the creation of this article.
